# *Cronobacter sakazakii* Infection from Expressed Breast Milk, Australia

**DOI:** 10.3201/eid2402.171411

**Published:** 2018-02

**Authors:** Rowena McMullan, Vidthiya Menon, Alicia G. Beukers, Slade O. Jensen, Sebastiaan J. van Hal, Rebecca Davis

**Affiliations:** Royal Prince Alfred Hospital Women and Babies, Sydney, New South Wales, Australia (R. McMullan);; Royal Prince Alfred Hospital, Sydney (V. Menon, A.G. Beukers, S. van Hal, R. Davis);; Western Sydney University, Sydney (S.O. Jensen)

**Keywords:** *Cronobacter sakazakii*, meningitis/encephalitis, expressed breast milk, neonates, bacteria, bacterial infections, Australia

## Abstract

*Cronobacter sakazakii* neonatal infections are often epidemiologically linked to the consumption of contaminated powdered infant formula. We describe a case resulting from consumption of contaminated expressed breast milk, as confirmed by whole-genome sequencing. This case highlights potential risks associated with storage and acquisition of expressed breast milk.

*Cronobacter sakazakii* neonatal infections can cause severe systemic infection and meningitis, resulting in mortality rates as high as 42% (*1*). *C. sakazakii* infections have been epidemiologically linked with contaminated powdered infant formula (PIF), whereas reports of *Cronobacter* infection in infants exclusively fed breast milk are rare (*1*). In 2016, a case of clinical meningitis was reported in an infant who had consumed expressed breast milk (EBM) contaminated with *C. sakazakii* (*2*). The source of contamination was unknown; however, pulsed-field gel electrophoresis revealed indistinguishable isolates from a contaminated breast pump and EBM. We report a similar case of an infant with onset of *C. sakazakii* clinical meningitis after consumption of contaminated EBM. We confirmed the source of the infection by using whole-genome sequencing (WGS).

In 2015, a 30-year-old woman underwent preterm labor at 27 weeks and 5 days and delivered a male infant. Cultures of infant blood specimens collected soon after birth were negative for bacteria and fungi. From day 2 of life, the infant received probiotics (Infloran; Laboratorio Farmaceutico, Mede, Italy) and was fed exclusively with EBM administered through an orogastric feeding tube. On day 10 of life, the infant’s health suddenly deteriorated, requiring intubation and ventilation. Blood cultures grew mucoid yellow colonies that we identified as *C. sakazakii* by using matrix-assisted laser desorption/ionization time-of-flight mass spectrometry (Bruker Daltonics, Breman, Germany). Despite appropriate antimicrobial treatment with meropenem, the infant had onset of status epilepticus, pulmonary hemorrhage, and acute renal failure. After discussion with his parents, care was redirected to palliation, and the infant died at 11 days of age.

Samples of EBM stored on the neonatal unit at 4°C were sent for culture. Two milk samples expressed during the mother’s 7-day inpatient stay were cultured and grew skin flora. Three samples expressed during the 6 days after discharge grew *C. sakazakii*. After leaving the hospital, the mother expressed breast milk by using a handheld breast pump that had not been sterilized before use. EBM was brought to the unit and stored in the same manner as EBM expressed while in hospital. 

We conducted WGS on isolates of *C. sakazakii* cultured from EBM and the infant’s blood (Technical Appendix). The EBM and infant isolates were identical, with 6 single-nucleotide polymorphisms between them, confirming that the infant was exposed to the pathogen through consumption of EBM (Figure).

**Figure:**
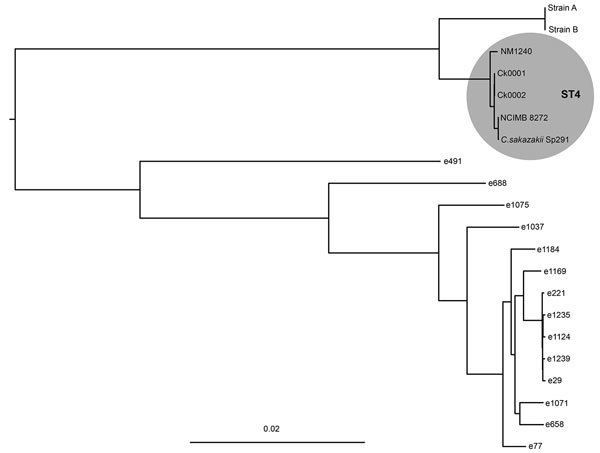
Maximum-likelihood phylogeny of *Cronobacter* isolates cultured from the blood of an infant (Ck0001) and the mother’s expressed breast milk (Ck0002) with *C. sakazakii* Sp291 as reference. Shaded circle highlights the clustering of sequence type 4 isolates. Scale bar indicates nucleotide substitutions per site. Methods for culturing isolates described in Technical Appendix. ST, sequence type.

*C. sakazakii* has been shown to colonize equipment used to prepare and administer milk formula (*3*). The risks associated with consumption of PIF and *Cronobacter* infection in infants are well understood. Consequently, much effort has gone into providing safe instructions and guidelines for preparation and storage of PIF to prevent such infections, including appropriate cleaning and sterilization procedures and storage conditions for this heat-resistant organism. *C. sakazakii* have been shown to survive and grow in human breast milk at temperatures of 10°C, 23°C, and 37°C (*4*) after introduction of the organism from an external source. Therefore, in the case of our neonate patient, the handheld breast pump probably was colonized with *C. sakazakii*, leading to contamination of the EBM (especially because EBM cultures while in hospital were negative for *Cronobacter*) and subsequent infection.

Per hospital practice at the time of this case, mothers who were inpatients and expressing breast milk were advised to perform hand hygiene before using or cleaning the hospital breast milk pump kits. The kits were washed in hot soapy water, rinsed and dried after use, and sterilized every 24 hours. After discharge from the hospital, mothers were to use their own reusable kits and breast pumps and were given the same cleaning advice about the kits. In this case, it appears that although verbal and written advice was given initially, no follow-up discussion occurred, and a pump was used without sterilization of the kit. Subsequently, several changes have been instituted, including processes to ensure daily discussion with mothers about breastfeeding and breast milk hygiene, especially given that parents of preterm infants are often in an unexpected and highly stressful situation, when information retention is difficult. Women are also advised to rent or buy a breast pump rather than borrow a pump.

Unfortunately, the risks associated with EBM are not well recognized. This fact is becoming increasingly important because globally an increasing number of premature infants are cared for on neonatal units and require EBM until feeding is established. This case and others of *Cronobacter* isolation from EBM or contaminated expressing equipment suggest that consumption of contaminated EBM might be more common than initially thought, highlighting the importance of education to new parents who will be expressing breast milk for their infants. Recommendations by the US Centers for Disease Control and Prevention include correct sanitation procedures to clean breast pumps, safe storage techniques between breast pump use, and safe storage of EBM (*5*). If infants are unable to feed directly at the breast, reducing exposure of EBM to environmental organisms through appropriate care of equipment is essential to maintain the safety of this vital source of nutrition.

Technical Appendix Methods for culturing *Cronobacter* isolates from the infant’s blood and the mother’s expressed breast milk.
