# Supplementary material for: Cronobacter sakazakii Infection from Expressed Breast Milk, Australia
Source: Emerg Infect Dis. 2018 Feb;24(2):393–4. doi: 10.3201/eid2402.171411 (PMC5782911; doi:10.3201/eid2402.171411)
Supplement: Technical Appendix — Methods for culturing Cronobacter isolates from the infant’s blood and the mother’s expressed breast milk. [file 17-1411-Techapp-s1.pdf]

Article DOI: https://doi.org/10.3201/eid2402.171411

Cronobacter sakazakii Infection from Expressed Breast Milk, Australia

Technical Appendix

Supplemental Information

The genomic DNA of *C. sakazakii* isolates grown on horse blood agar (HBA) at 37°C for 24 h were extracted and libraries were prepared using the Nextera XT DNA Library Preparation Kit (Illumina, San Diego, California). The isolates were sequenced on an Illumina MiSeq (Illumina) using the Miseq v2 Micro Kit (150bp and 1.2Gb output; Illumina) at the Ramaciotti Centre for Genomics. These isolates were supplemented with available *C. sakazakii* illumina sequence reads downloaded from the sequenced read archive (SRA) available from NCBI and included for analysis (Technical Appendix Table). Sequencing reads for each isolate were mapped to the finished genome of *C. sakazakii* SP291 (GenBank accession CP004091) using BWA (*1*). Variants were called using FreeBayes (v1.0.2-dirty) and filtered based on mapping quality, base quality, coverage (minimum 10) and allele frequency of greater than >90% (*2*). Insertions and deletions were excluded from the analysis. A maximum likelihood (ML) phylogenetic tree was constructed using FastTree (version 2.1.8) (*3*), using a generalized time-reversible model and manipulated in FigTree (version 1.4.2) (*4*). Identified variants were annotated using snpEff (version 4.3i) (*5*) with SP291 as the reference. Hypothetical regions were re-annotated using prokka and blastn to include up to date data. All variants were subsequently mapped to branches on the phylogeny using an in-house script.

References

1. Li H, Durbin R. Fast and accurate short read alignment with Burrows-Wheeler transform. Bioinformatics. 2009;25:1754–60. PubMed http://dx.doi.org/10.1093/bioinformatics/btp324

2. Garrison E, Marth G. Haplotype-based variant detection from short-read sequencing [cited 2017 Apr 11]. https://arxiv.org/abs/1207.3907

3. Price MN, Dehal PS, Arkin AP. FastTree 2—approximately maximum-likelihood trees for large alignments. PLoS One. 2010;5:e9490. PubMed http://dx.doi.org/10.1371/journal.pone.0009490

4. Rambaut A. Molecular evolution, phylogenetics and epidemiology—FigTree [cited 2017 Apr 11]. http://tree.bio.ed.ac.uk/software/figtree

5. Cingolani P, Platts A, Wang L, Coon M, Nguyen T, Wang L, et al. A program for annotating and predicting the effects of single nucleotide polymorphisms, SnpEff: SNPs in the genome of *Drosophila melanogaster* strain w1118; iso-2; iso-3. Fly (Austin). 2012;6:80–92. PubMed http://dx.doi.org/10.4161/fly.19695

Technical Appendix Table. Isolates used in genomic analysis*

Strain ID	ST	Source	Isolation date	Country	Submitter	Accession no.
C. sakazakii Sp291	4	PIF manufacturing environment	2013	Ireland	University College Dublin	NC_020260
Ck0001	4	Blood of infant	2015	Australia	Royal Prince Alfred Hospital	SAMN06919901
Ck0002	4	Expressed breast milk	2015	Australia	Royal Prince Alfred Hospital	SAMN06919902
NCIMB 8272	4	Milk powder	1950	UK	Nottingham Trent University	SRR944696
NM1240	4	Cerebrospinal fluid	2008	USA	FDA – CDC	SRR1814236
Strain A	8	Unknown	Unknown	Unknown	GIFU_MED	DRR015812
Strain B	8	Unknown	Unknown	Unknown	GIFU_MED	DRR015984
e29	415	Unknown	Unknown	Unknown	Sanger Institute	ERR474280
e1037	416	Unknown	Unknown	Unknown	Sanger Institute	ERR474430
e1071	417	Unknown	Unknown	Unknown	Sanger Institute	ERR474434
e1075	418	Unknown	Unknown	Unknown	Sanger Institute	ERR474435
e1124	415	Unknown	Unknown	Unknown	Sanger Institute	ERR474436
e1169	419	Unknown	Unknown	Unknown	Sanger Institute	ERR474449
e1184	420	Unknown	Unknown	Unknown	Sanger Institute	ERR474450
e1235	415	Unknown	Unknown	Unknown	Sanger Institute	ERR474458
e1239	415	Unknown	Unknown	Unknown	Sanger Institute	ERR474461
e77	421	Unknown	Unknown	Unknown	Sanger Institute	ERR486105
e221	415	Unknown	Unknown	Unknown	Sanger Institute	ERR486111
e658	422	Unknown	Unknown	Unknown	Sanger Institute	ERR486181
e688	423	Unknown	Unknown	Unknown	Sanger Institute	ERR486184
e491	424	Unknown	Unknown	Unknown	Sanger Institute	ERR502554

*ST, sequence type.
